# Supplementary material for: Crawling and Gliding: A Computational Model for Shape-Driven Cell Migration
Source: PLoS Comput Biol. 2015 Oct 21;11(10):e1004280. doi: 10.1371/journal.pcbi.1004280 (PMC4619082; doi:10.1371/journal.pcbi.1004280)
Supplement: S1 Code — (ZIP) [file pcbi.1004280.s012.zip › release/tst/doc/html/globals_func.html]

Tissue Simulation Toolkit: File Members


Tissue Simulation Toolkit
 0.1.4.1


- Main Page
- Namespaces
- Classes
- Files

- File List
- File Members

- All
- Functions
- Variables
- Typedefs
- Macros

- a
- b
- c
- d
- e
- f
- g
- h
- i
- m
- n
- o
- p
- r
- s
- t
- w
- y

### - a -

- AskSeed()
  : random.cpp
  , random.h

### - b -

- bgetpar()
  : parse.cpp
  , parse.h
- bool\_str()
  : parse.cpp
  , parse.h

### - c -

- CanWeWriteP()
  : output.h
- chainHull\_2D()
  : hull.cpp
  , hull.h
- CheckFile()
  : output.cpp
  , output.h
- Chext()
  : output.cpp
  , output.h
- conrec()
  : conrec.cpp
  , conrec.h
- Crash()
  : crash.cpp
  , crash.h

### - d -

- dgetparlist()
  : parse.cpp
  , parse.h

### - e -

- error()
  : warning.cpp
  , warning.h

### - f -

- fgetpar()
  : parse.cpp
  , parse.h
- FileExists()
  : misc.cpp
  , misc.h
- FileExistsP()
  : output.cpp
  , output.h

### - g -

- GetFileName()
  : misc.cpp
  , misc.h

### - h -

- HandleSIGINT()
  : crash.cpp
  , crash.h
- HandleSIGSEGV()
  : crash.cpp
  , crash.h

### - i -

- igetpar()
  : parse.cpp
  , parse.h
- isLeft()
  : hull.cpp

### - m -

- MakeDir()
  : output.h
- MemoryWarning()
  : crash.cpp
  , crash.h

### - n -

- NiceMessage()
  : crash.cpp
  , crash.h

### - o -

- OpenFileAndCheckExistance()
  : output.cpp
  , output.h
- OpenGZippedWriteFile()
  : output.h
- OpenReadFile()
  : output.cpp
  , output.h
- OpenWriteFile()
  : output.cpp
  , output.h
- operator<<()
  : parameter.cpp
  , parameter.h

### - p -

- ParsePar()
  : parse.cpp
  , parse.h

### - r -

- RANDOM()
  : random.cpp
  , random.h
  , sticky.h
- Randomize()
  : random.cpp
  , random.h
- RandomNumber()
  : random.cpp
  , random.h
- ReadDouble()
  : misc.cpp
  , misc.h
- ReadLine()
  : output.cpp
  , output.h
- ReadNumber()
  : misc.cpp
  , misc.h

### - s -

- sat()
  : ca.cpp
- sbool()
  : parameter.cpp
  , parameter.h
- SearchToken()
  : parse.cpp
  , parse.h
- Seed()
  : random.cpp
  , random.h
- sgetpar()
  : parse.cpp
  , parse.h
- SkipLine()
  : parse.cpp
  , parse.h
- SkipToken()
  : parse.cpp
  , parse.h
- StartSIGINTHandling()
  : crash.cpp
  , crash.h
- StartSIGSEGVHandling()
  : crash.cpp
  , crash.h

### - t -

- TokenInLineP()
  : parse.cpp
  , parse.h

### - w -

- warning()
  : warning.cpp
  , warning.h

### - y -

- YesNoP()
  : misc.cpp
  , misc.h
  , output.cpp
  , output.h


---

Generated on Thu Aug 14 2014 22:04:02 for Tissue Simulation Toolkit by  

 1.8.6
